# Current-induced shuttlecock-like movement of non-axisymmetric chiral skyrmions

**DOI:** 10.1038/s41598-019-56791-3

**Published:** 2020-01-15

**Authors:** Remi Murooka, Andrey O. Leonov, Katsuya Inoue, Jun-ichiro Ohe

**Affiliations:** 1 Department of Physics, Toho University, 2-2-1 Miyama, Funabashi, Chiba Japan; 2 Chirality Research Center, Hiroshima University, Higashi-Hiroshima, Hiroshima 739-8526 Japan; 3 Department of Chemistry, Faculty of Science, Hiroshima University Kagamiyama, Higashi Hiroshima, Hiroshima 739-8526 Japan; 4 IFW Dresden, Postfach 270016, D-01171 Dresden, Germany

**Keywords:** Physics, Condensed-matter physics, Spintronics

## Abstract

Current-induced motion of non-axisymmetric skyrmions within tilted ferromagnetic phases of polar helimagnets with the easy plane anisotropy is studied by micromagnetic simulations. Such non-axisymmetric skyrmions consist of a circular core and a crescent-shaped domain-wall region formed with respect to the tilted surrounding state. Current-driven motion of non-axisymmetric skyrmions exhibits two distinct time regimes: initially the skyrmions rotate towards the current flow direction and subsequently move along the current with the skyrmionic crescent first. According to the Thiele equation, the asymmetric distribution of the topological charge and the dissipative force tensor play an important role for giving the different velocities for the circular and the crescent-shaped constituent parts of the skyrmion what underlies such a shuttlecock-like movement. Moreover, the current-velocity relation depends on the angle of the tilted ferromagnetic phase what makes in particular the transverse velocity of skyrmions sensitive to their field-driven configurational transformation. We also argue the possibility of magnetic racetrack waveguides based on complex interplay of robust asymmetric skyrmions with multiple twisted edge states.

## Introduction

Magnetic chiral skyrmions are topological excitations with particle-like properties that have complex non-coplanar spin structure^1–5^. Skyrmions were recently discovered in bulk non-centrosymmetric helimagnets^6–10^ and in nanostructures with confined geometries over larger temperature regions^11–13^. According to general arguments by Hobart and Derrick^14^, however, any multidimensional localized states are unstable in many physical field models: particle-like inhomogeneous states may arise only as dynamic excitations, but static configurations are unstable and collapse into topological singularities^14^. Consequently, non-linear field equations produce only one-dimensional soliton-like solutions. Chiral Dzyaloshinskii-Moriya interactions (DMI)^15^ represent a distinct stabilization mechanism working against the constraints of the Hobart-Derrick theorem^14^ and thus protects chiral skyrmions from radial instability^1,16^. As a result, non-centrosymmetric magnets constitute a particular class of materials where skyrmions can exist and thus are of special interest in fundamental physics and mathematics^17,18^. Nanometer size of chiral skyrmions, their topological stability and manipulation by electric currents^19–21^ enabled a new burgeoning field of research in non-volatile memory and logic devices^22,23^. In particular, in the skyrmion racetrack^23–25^ – a probable model for future information technology – the flow of information is encoded in the moving metastable skyrmionic bits^26^.

The customary approach to enhance the functionality of the skyrmion-based racetrack memory is the mechanical patterning of underlying nanosamples. As an example, suggested devices may feature a regular arrangement of notches to divide the track into a sequence of parking lots for the skyrmions^26,27^. An additional nanostrip on top of the racetrack may create an energy barrier along the middle and thus forms two channels for a skyrmion movement^24^. This enables the information storage in the lane number of each skyrmion. Moreover by adding stripes with high magnetic crystalline anisotropy at the edges, one confines the skyrmions inside and prevents their escape from the nanotrack^28^. One should also mention elaborate schemes that include, e.g., Y-shaped junctions^22,23,29^. By these, the skyrmions can be selectively driven into different nanotracks and form complementary data representation. Moreover, spin logic gates such as the “AND” and “OR” operations based on manipulations of skyrmions can be designed^22,23,29^.

**Figure 1:**
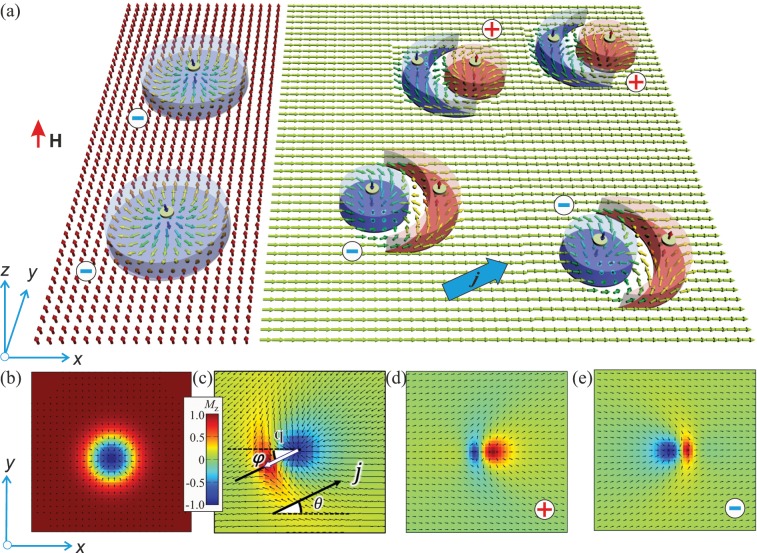
(**a**) Schematic of the current-induced motion of isolated skyrmions in polar magnets. Magnetic field is applied along *z*-axis and either saturates the surrounding state (left) or due to the competition with the easy-plane anisotropy leads to the angular phase (right). Skyrmions implanted into these homogeneous states have either axisymmetric (**b**) or non-axisymmetric (**c**) shapes. On the contrary to the saturated state that hosts skyrmions only with the negative polarity (**b**), the angular phase accommodates both types of NIS with the magnetization in their cores both along and opposite to the field (**d**,**e**). Note the opposite orientation of the crescent with respect to the cores with opposite polarity. The color plots indicate *z*-component of the magnetization in AIS (**b**) and NIS with positive (**d**) and negative (**e**) polarities. Black arrows are projections of the magnetization on to the *xy* plane. The spin polarized current comprises angle *θ* with *x*- axis while NIS-dipoles **q** – angle *φ* as shown in (**c**).

An alternative approach to enhance the functionality of the skyrmion-based racetrack memory is to utilize the unique properties of non-axisymmetric isolated skyrmions (NISs) that in particular may emerge within tilted ferromagnetic (TFM) phases of polar magnets with the easy-plane anisotropy^30^ (Fig. 1(a), right). TFM state represents a homogeneously magnetized state with a ferromagnetic moment tilting away from the polar axis due to the competition between the easy-plane anisotropy and the field. As compared with the ordinary axisymmetric isolated skyrmions (AISs) within the field-saturated state^16^ (left side of Fig. 1(a,b)), NIS may acquire both polarities in their cores (although the vorticity bears the same sign) and thus are subject to the skyrmion Hall effect with opposite shift directions (and hence naturally form two channels, Fig. 1(d,e)). Based on the opposite sign of the topological charge, one may call two types of NISs – skyrmions (Fig. 1(d)) and anti-skyrmions (Fig. 1(e)), and consider them as binary data bits for possible practical applications. Note that skyrmions and antiskyrmions with the same polarity but the opposite vorticity were recently investigated in frustrated magnets with competing exchange interactions^31–33^.

In the present paper, we explore the current-induced dynamics of introduced non-axisymmetric skyrmions. We show that depending on the direction of the spin-polarized current (SPC) with respect to the skyrmion orientation, NIS undergoes a rotation towards the SPC with its subsequent current-aligned movement. The velocity of NIS is effectively regulated by the field magnitude (and thus by the tilt angle of the surrounding angular phase). We also underline prospects of using NISs in racetrack memory devices. Anisotropic skyrmion-skyrmion interaction that depends on their mutual orientation^30^ alongside with the three types of edge states naturally formed at the lateral edges of a racetrack, make NISs effective candidates to be employed in nanoelectronic devices of the next generation in which nanopatterning is boiled down to a minimum.

## Micromagnetic Model

The equilibrium solutions for NIS are derived within the standard discrete model of a polar helimagnet^9,10,30^ where the total energy is given by:

1 $$\begin{array}{rcl}w & = & J\,\sum _{ < i,j > }\,({{\bf{M}}}_{i}\cdot {{\bf{M}}}_{j})-\sum _{i}\,{\bf{H}}\cdot {{\bf{M}}}_{i}-K{M}_{z}^{2}\\  &  & -\,D\,\sum _{i}\,({{\bf{M}}}_{i}\times {{\bf{M}}}_{i+\hat{x}}\cdot \hat{y}-{{\bf{M}}}_{i}\times {{\bf{M}}}_{i+\hat{y}}\cdot \hat{x}\mathrm{)}.\end{array}$$

**Figure 2:**
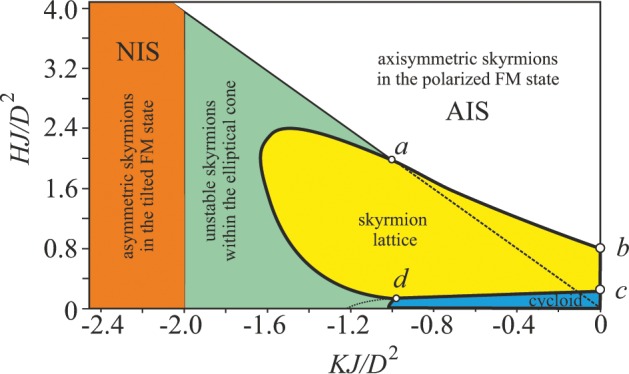
Magnetic phase diagram of the solutions for model (1) with the easy-plane anisotropy. Filled areas designate the regions of thermodynamical stability of corresponding phases: blue shading - cycloidal spiral, green shading - elliptical cone, white shading - polarized ferromagnetic state, yellow shading - hexagonal skyrmion lattice, orange shading - tilted ferromagnetic state. Thick black lines indicate the first-order phase transitions between corresponding phases, thin black lines - the second-order phase transitions.

**M**_*i*_ is the unit vector in the direction of the magnetization at the site *i* of a two-dimensional square lattice and <*i*, *j*> denote pairs of nearest-neighbor spins. $\hat{x}$ and $\hat{y}$ are unit vectors along *x* and *y* directions, respectivel*y*. The first term describes the ferromagnetic nearest-neighbor exchange with *J* < 0, the second term is the Zeeman interaction with the magnetic field parallel to the *z* axis, and the third term is the easy-plane anisotropy with *K* < 0. Throughout the paper, we use the value of *K* that enables only TFM formation, i.e. we omit the regions of the *H* − *K* phase diagram that host modulated skyrmion lattice (SkL), spiral and elliptical cone phases^34^ (Fig. 2). Dzyaloshinskii-Moriya interaction (DMI) stabilizes NISs with the Néel type of the magnetization rotation. The DMI constant *D* = *J*tan(2*π*/*p*) defines the characteristic size of skyrmions with the period of modulated structures *p*. In the following simulations, *D* is set to 0.5*J*. Within model (1), AISs exist as metastable excitations of the saturated state for *H* > *H*_*cr*_ = 2*K* (Fig. 1(b)), while NISs (Fig. 1(c)) are present for lower fields. We use *K* = −2.6*D*^2^/*J* to consider metastable NISs, and hence *H*_*cr*_ = 5.2*D*^2^/*J* (Fig. 2).

AIS are characterized by azimuthal (*θ*) and polar (*ψ*) angles of the spins according to $\theta =\theta (\rho ),\,\psi =\varphi$. Here the boundary conditions are *θ*(0) = *π*, *θ*(∞) = 0, while *φ* and *ρ* are cylindrical coordinates of the spatial variable (Fig. 1(a), left). On the other hand, NISs are confined by the following in-plane boundary conditions: *θ*(0) = *π*, *θ*(∞) = *θ*_*TFM*_ = arccos(*H*/2*K*). These boundary conditions violate the rotational symmetry, forcing the skyrmions to develop an asymmetric shape (Fig. 1(a), right).

The complete phase diagram (Fig. 2) of states of the model (1) has been reproduced from refs. ^30,34^ and includes stability regions of modulated phases and regions of metastable skyrmions. The phase diagram also allows to generalize the processes of skyrmion lattice formation. Along the line *a* − *b* the skyrmion lattice appears as a result of condensation of isolated skyrmions (building blocks of the hexagonal skyrmion lattice), as found for axisymmetric skyrmions in the easy-axis case^1,4,16^. Along the line *c* − *d*, hexagonal skyrmion lattice may appear as a result of local cutting of the cycloid (in this sense, two merons may be considered as nuclei of the skyrmion lattice. Along the first-order phase transition line *a* − *d*, however, none of the aforementioned mechanisms is appropriate. Presumably, domains of the skyrmion lattice and the elliptical cone state coexist with non-trivial domain boundary between them.

**Figure 3:**
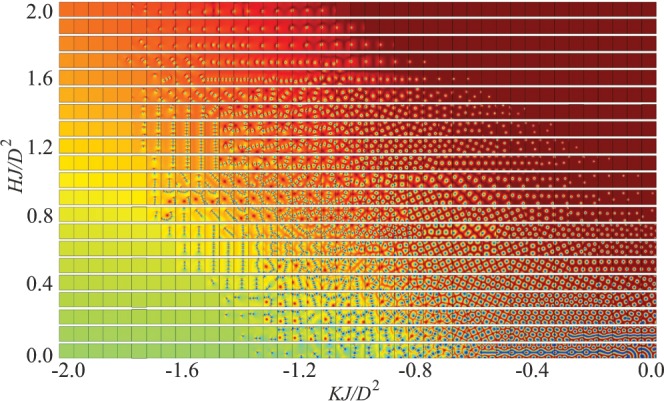
Magnetic structure of solutions on the plane (magnetic field)– (easy-plane anisotropy). Such an alternative phase diagram could be rather applied to magnetic nanostructures with confined geometries (see text for details).

Figure 3 exhibits the magnetic structure of all the states from the phase diagram in Fig. 1. The states in Fig. 2 include in particular skyrmion chains, disordered glassy states of NISs, as well as square arrangements of AISs. We, however, state that additional minimization with respect to the size of considered numerical grids preserves only the phases from the phase diagram in Fig. 2. All other phases are the result of imposed confinement and thus could be realized in nanostructures with confined geometries.

In the present manuscript, however, we consider only metastable NISs surrounded by the TFM state (orange shaded region in Fig. 2) what is required for racetrack memory devices. We avoid regions of the phase diagram where skyrmions form skyrmion lattices or undergo elliptical instability, as well as the regions of one-dimensional spiral states.

The current-driven dynamics of NISs and AISs was simulated using Landau-Lifshitz-Gilbert (LLG) equation^35,36^:

2 $$\frac{d{{\bf{M}}}_{i}}{dt}=-\,\gamma {{\bf{M}}}_{i}\times {{\bf{H}}}_{{\rm{eff}}}+\frac{\alpha }{{M}_{{\rm{s}}}}{{\bf{M}}}_{i}\times \frac{d{{\bf{M}}}_{i}}{dt}+\tau +{\tau }_{\beta }.$$

Here *γ* is the gyromagnetic ratio and *α* = 0.01 is the Gilbert damping constant. This value of *α* is common and widely used in skyrmionics. The value of the same order, *α* = 0.04 or even smaller *α* = 0.004, have been used to theoretically study spin-wave modes and their intense excitations activated by microwave magnetic fields in the SkL phases of insulating magnets^37^. *α* = 0.04 is a typical value for the ferromagnetic metal and the dilute magnetic semiconductors^38^. *α* = 0.01 was used to address the dynamics of skyrmions in frustrated magnets^31,33^. Since the velocity of AISs is inversely proportional to *α* and the SPC-velocity characteristics in the SkL phase remain universal, independent on *α*, nonadiabatic effect *β* and impurities^38^, the substantial room for improvement of skyrmion velocity is provided, because many magnetic materials show *α* much smaller than that for cobalt^26^, *α* = 0.3.

*H*_eff_ is a local effective magnetic field, which at the site *i* is given by **H**_eff_ = −∂*w*/∂**M**_*i*_. The spin transfer torque (STT) consists of the adiabatic part, *τ* = *A*(**j** ⋅ ∇)**M**_*i*_, and the non-adiabatic term, *τ*_*β*_ = *Aβ***M**_*i*_ × (**j** ⋅ ∇)**M**_*i*_, where *A* = *Jgμ*_*B*_/2*eM*_*s*_ is the coefficient proportional to the SPC density. The SPC **j** comprises an angle *θ* with *x*-axis (Fig. 1(c)). An orientation of NIS is characterized by an angle *φ* between a vector, which connects centers of the circular core and the crescent (skyrmion dipole **q**), and *x*-axis (Fig. 1(c)).

## Current-Induced Motion (A Shuttlecock-Like Movement)

**Figure 4:**
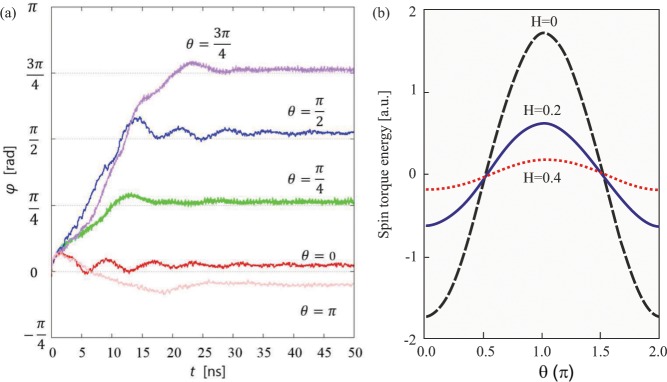
(**a**) Current-induced rotation of NISs. Independently on the angle *θ*, the NIS-dipoles acquire the same orientation angle *φ*, i.e. become co-aligned with the SPC direction. Such a forced rotation is explained by the STT energy (**b**) that is minimized only for the current-aligned movement.

To systematically investigate the current-driven dynamics of NISs, we applied the SPC with different angles with respect to the skyrmion dipole initially oriented with $\varphi =0$ (Fig. 4(a)). It was found that after an initial rotation towards the SPC, NIS-dipoles are always current-aligned with $\varphi =\theta$ (Fig. 4(a)). A relatively small increment of the SPC angle *θ* allowed to exclude local minima of the skyrmion orientation with respect to the SPC. In particular, a NIS motion with its core along the SPC was excluded (although in a numerical experiment of Fig. 4(a) such a movement opposite to the SPC appeared to be feasible).

### Rotation of NIS towards the SPC direction

Figure 4(a) shows the time dependence of the dipole angle $\varphi$ for different SPC directions *θ*. As expected, more time is needed to orient NIS along the SPC with increasing angle *θ*. The skyrmion rotation originates from the non-axisymmetric internal structure. By applying the charge current, the magnetization structure is modulated by the spin transfer torque. This modulation changes the total energy of the magnetic system. The energy gain due to the spin transfer torque *E*^STT^ can be expressed as ${E}^{{\rm{STT}}}={\int }_{{\rm{sk}}}\,d{\bf{r}}{{\bf{M}}}_{i}\cdot {{\bf{H}}}_{{\rm{eff}}}$. Figure 4(b) shows the *E*^STT^ as a function of *θ* featuring minima only along the SPC. We use the magnetic configuration of *t* = 10^−13^ *s* after the current is applied. The minima, however, become shallow with the increasing magnetic field and completely disappear for *H* > *H*_*cr*_ with the onset of the field-saturated state.

### Translational movement of NISs

The current-induced translational motion of NIS is well understood in terms of the Thiele equation^39^

3 $${\bf{G}}\times ({\bf{j}}-{\bf{v}})+\boldsymbol{\mathscr{D}}(\beta {\bf{j}}-\alpha {\bf{v}})=\nabla V,$$

where **v** is the velocity of the skyrmion and *V* is the pinning potential. The gyromagnetic coupling vector **G** = (0, 0, *G*_*z*_) equals the topological charge

4 $${G}_{z}=\frac{1}{4\pi }\int \,d{\bf{r}}\frac{1}{{M}_{{\rm{s}}}^{3}}{\bf{M}}\cdot ({\partial }_{x}{\bf{M}}\times {\partial }_{y}{\bf{M}})\mathrm{}.$$

$\boldsymbol{\mathscr{D}}$ is the dissipative force tensor:

5 $${\boldsymbol{\mathscr{D}}}_{ij}=\frac{1}{2\pi }\int \,d{\bf{r}}\frac{1}{{M}_{{\rm{s}}}^{2}}{\partial }_{i}{\bf{M}}\cdot {\partial }_{i}{\bf{M}}\,(i,j=x,y).$$

which is not symmetric for NISs $({\boldsymbol{\mathscr{D}}}_{xx}\ne {\boldsymbol{\mathscr{D}}}_{yy})$, even the off-diagonal element has a finite value $({\boldsymbol{\mathscr{D}}}_{xy}\ne \mathrm{0)}$. Thus, when the charge current **j** is applied, the skyrmion feels both the longitudinal and the Magnus forces^38^.

For *θ* = 0, the longitudinal (*v*_*x*_) and the transverse (*v*_*y*_) components of the velocity are represented as

6 $${v}_{x}=\frac{{G}_{z}^{2}+{D}_{xx}{D}_{yy}\alpha \beta +\xi \beta {D}_{yx}}{{G}_{z}^{2}+{D}_{xx}{D}_{yy}{\alpha }^{2}+\xi \alpha {D}_{xy}}j,$$

7 $${v}_{y}=\frac{(\alpha -\beta ){D}_{xx}{G}_{z}}{\xi \{{G}_{z}^{2}+{D}_{xx}{D}_{yy}{\alpha }^{2}-\xi \alpha {D}_{xy}\}}j\mathrm{}.$$

where *ξ* = *G*_*z*_ − *αD*_*xy*_.

**Figure 5:**
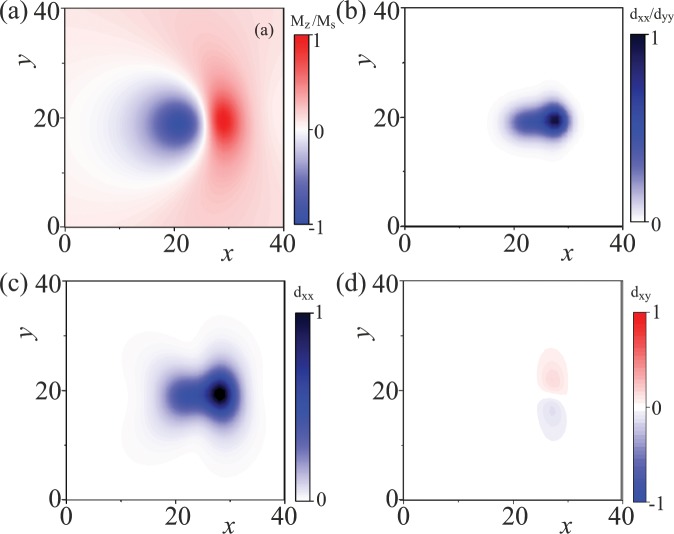
Color plots of the *M*_*z*_ component and the dissipative force tensor *d*_*xx*_*d*_*yy*_ (**b**), *d*_*xx*_ (**c**) and *d*_*xy*_ (**d**) as defined by Eq. (5). The depicted distributions account for a rotational movement of NIS as well as for the translational dynamics in Fig. 6 (see text for details).

**Figure 6:**
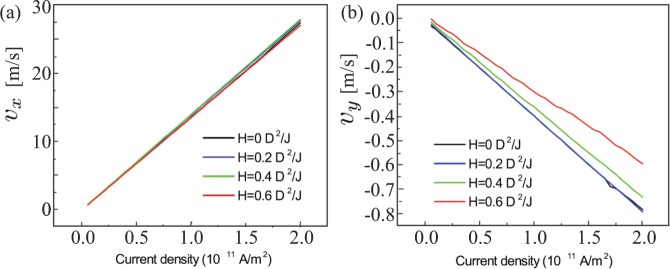
Longitudinal *v*_*x*_ (**a**) and transversal *v*_*y*_ (**b**) velocities of the current-induced motion of NISs for several values of the applied magnetic field and therefore for different TFM states. Whereas *v*_*x*_ is clearly field-independent what complies with the Thiele Eq. (3), *v*_*y*_ strongly depends on the skyrmion internal structure (see text for details).

We plot the spatial configuration of the dissipative force tensor in Fig. 5(b–d). With a sufficiently large value of *d*_*xx*_*d*_*yy*_ = (∂_*x*_**M**⋅∂_*x*_**M**)(∂_*y*_**M**⋅∂_*y*_**M**) (plotted in Fig. 5(b)), *v*_*x*_ = *β*/*α*, which is the same as for AISs^38^ and is consistent with the universal *j* − *v*_*x*_ relation independent of *β* (Fig. 6(a)). The transverse velocity, on the contrary, is proportional to *d*_*xx*_ (Fig. 5(c)) and is strongly field-dependent (Fig. 6(b)), since the field affects the spin configuration of NISs, which also may be reflected in the skyrmion Hall angle of the NIS. One can also see that *d*_*xx*_*d*_*yy*_ of the crescent part is larger than that of the circular part (Fig. 5(a,b)). This asymmetry of *d*_*xx*_*d*_*yy*_ underlies the faster velocity of the crescent resulting in the rotational motion and is the reason why we dubbed such a motion “a current-induced shuttlecock-like movement”. We neglect the effect of the off-diagonal element of the dissipative force tensor *d*_*xy*_ = ∂_*x*_**M**⋅∂_*y*_**M**, since it is much smaller than *d*_*xx*_ and has opposite sign for upper and lower parts of NISs (Fig. 5(d)).

## Edge States

**Figure 7:**
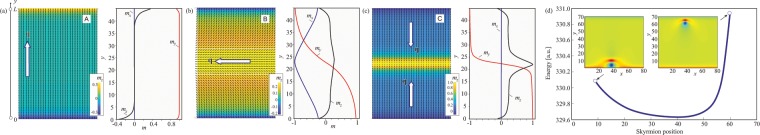
(**a–c**) Edge states in a nanostripe with the easy-plane anisotropy. An applied magnetic field induces consecutive phase transitions A-B-C (see text for details). (**d**) The total energy of the magnetic system (insets) in which the NIS is set on the racetrack. The position of the NIS is defined as the center of the circular vortex part. The total energy becomes larger when the NIS comes near to the edges that assures the propagation of the NISs in the quasi-one dimensional racetrack waveguide.

The practical use of NISs in racetracks hinges on their interaction with edge states. For racetracks with the field-saturated magnetization, the edge states manifest themselves as remnants of the helical spiral and repulse AISs^40^. For racetracks with the TFM state, however, three different types of edge states can arise (in Fig. 7 the edge states are marked with capital letters A, B, and C).

### Transitions between edge states

The type A edge state with collinear in-plane spin components (azimuthal angle of the magnetization is *ψ* = *π*/2) is induced for lower values of the applied magnetic field. *z*-component of the magnetization acquires opposite signs at the opposite edges which leads to an asymmetric NIS-edge interaction potential (Fig. 7(d)). With increasing magnetic field, the A-type edge state undergoes the first-order transition into the type B edge state with the rotating magnetization across the racetrack width *L*. Once *m*_*z*_ reaches unit value in the racetrack middle, the B-type edge state by the second-order transition transforms into the type C edge state with *ψ* = *π*/2 (Fig. 7(c)).

### Orientational confinement of NISs

Three different types of edge states formed at the lateral boundaries of the racetrack also impose an orientational confinement on non-axisymmetric skyrmions. The A-type state implies perpendicular orientation of a skyrmion dipole **q** with respect to the racetrack edges (white arrow in Fig. 7(a) and magnetic configurations with NISs located near both stripe edges, Fig. 7(d)). At the same time due to the asymmetry of the magnetization distribution within the opposite edges, a NIS will be located closer to one edge than to the other (Fig. 7(d)). The SPC*j*_*x*_, by inducing the skyrmion rotation, may also initiate a transition A-B between edge states in spite of the B-type state is a metastable solution. The B-type state, on the contrary, may accommodate NISs with **q**||*x*. Thus, a moving NIS due to the skyrmion Hall effect will shift the whole stripe with the maximal *M*_*z*_-component (marked by the dashed lines in Fig. 7(b)) towards one of the edges. The C-type edge state allows two opposite NIS orientations degeneracy of which could be removed by the SPC *j*_*x*_ (Fig. 7(c)). In the present calculations, we avoid such extreme regimes under the larger current densities when (i) the skyrmions overcome the repulsive potential barrier from edge states what leads to skyrmion annihilation; (ii) the skyrmions are strongly deformed or even decay into magnons.

We also show that NISs rotate the surrounding homogeneous state, which otherwise is insensitive to SPC. Thus, NISs could be utilized as tumblers in nanostructures that rotate the surrounding oblique phases. In Supplementary Video, the current is applied perpendicular to the NIS-dipoles. After NISs have been rotated along the current, the current is switched off (*j* = 0 for *t* > 35 ns). This leads to the repulsive interaction between NISs^30^.

The considered effect is based on the anisotropic NIS-NIS interaction potential^30^: NISs attract each other being oriented head-to-head (initial configuration in Supplementary Video) and repulse being oriented side-to-side (the configuration after *t* = 35 ns). Thus, we stress that the SPC may disassemble the coupled pair of NISs or vice versa couples remote skyrmions into skyrmion chains. Recently, the chains of NISs were observed in chiral LC by the group of Ivan Smalyukh^41–43^.

## Conclusions

In conclusion, we examined the current-induced dynamics of non-axisymmetric skyrmions that exist within TFM states of *bulk* polar helimagnets with the easy plane anisotropy. In particular, uniaxial anisotropy of easy-axis and easy-plane type is attributed to the bulk polar magnetic semiconductors GaV_4_S_8_^9^ and GaV_4_Se_8_^10^ (the *C*_3*v*_ symmetry), respectively. Since the value of this effective anisotropy in these lacunar spinels is temperature-dependent, these material family establishes an ideal ground for the thorough study of anisotropic effects on modulated magnetic states^10^. The results obtained within the model (1) are also valid for *thin films* with interface induced DMI^44,45^.

We considered a shuttlecock-like movement of NISs that consists in their rotation to coalign with the SPC. We succeeded in modifying the current-velocity relation by the field-driven control of the angle in a surrounding homogeneous state. In ref. ^29^, conversion between skyrmions with axisymmetric and non-axisymmetric shape was achieved in a setup where the left input and right output wide regions with different material parameters are connected by a narrow nanowire. We remark that in ref. ^29^, the non-axisymmetric skyrmions are called bimerons. Such a terminology is also widely used to describe NISs in frustrated magnets^46,47^. Since frustrated magnets endow isolated skyrmions with the additional degrees of freedom - vorticity and helicity, easy-plane anisotropy forces AIS to transform into a bound pair of energetically equivalent merons with opposite vorticities, each carrying topological charge 1/2. In ref. ^46^, a meron cluster with a square lattice of vortices and antivortices was realized. Real-space observations of a two-dimensional square lattice of merons and antimerons emerging from a helical state was also reported in a thin plate of the chiral-lattice magnet Co _8_ Zn _9_ Mn _3_, which exhibits in-plane magnetic anisotropy^48^. In polar helimagnets, the pattern of DMI vectors stabilizes only one of the formed merons leading to a crescent-shaped deformation of the other meron. Nevertheless, such a bimeron preserves its summary topological charge 1. The NIS-NIS interaction, however, has an anisotropic character and can be either attractive or repulsive depending on the relative orientation of the NIS pair. Thus, instead of square arrangement of merons^46,48^, chiral NIS develop disordered metastable meron spin textures^49^.

We also speculate that a NIS placed into the racetrack memory with three different types of the edge states not only undergoes an orientational confinement, but can also be used as a current-activated tumbler between edge states. Our results are not only relevant to the application of magnetic skyrmions in memory technology but also elucidate the fundamental properties of skyrmions and the edge states formed in the TFM states of polar helimagnets^10,50^.

## Supplementary information

Supplementary information.

Supplementary information.
