# Supplementary material for: Current-induced shuttlecock-like movement of non-axisymmetric chiral skyrmions
Source: Sci Rep. 2020 Jan 15;10:396. doi: 10.1038/s41598-019-56791-3 (PMC6962387; doi:10.1038/s41598-019-56791-3)
Supplement: Supplementary file 2 — Supplementary information. [file 41598_2019_56791_MOESM2_ESM.pdf]

Supplementary Information for Current-induced shuttlecock-like movement of non-axisymmetric chiral skyrmions

Remi Murooka and Jun-ichiro Ohe*

Department of Physics, Toho University,

2-2-1 Miyama, Funabashi, Chiba, Japan

Andrey O. Leonov†

Chirality Research Center, Hiroshima University,

Higashi-Hiroshima, Hiroshima 739-8526, Japan

Department of Chemistry, Faculty of Science,

Hiroshima University Kagamiyama,

Higashi Hiroshima, Hiroshima 739-8526, Japan and

IFW Dresden, Postfach 270016, D-01171 Dresden, Germany

Katsuya Inoue‡

Chirality Research Center, Hiroshima University,

Higashi-Hiroshima, Hiroshima 739-8526, Japan and

Department of Chemistry, Faculty of Science,

Hiroshima University Kagamiyama,

Higashi Hiroshima, Hiroshima 739-8526, Japan

(Dated: August 22, 2019)

*junichirou.ohe@sci.toho-u.ac.jp

†leonov@hiroshima-u.ac.jp

‡kxi@hiroshima-u.ac.jp

I. SUPPLEMENTARY MOVIE

Simulated current-induced motion of non-axisymmetric skyrmions in an infinite 2D film with periodic boundary conditions. $H = 0.2D^2/J$, $K = 2.6D^2/J$. In the initial state we have a cluster of two NISs: skyrmions are positioned head-to-head according to the minimum of the interaction potential [27]. Color indicates m_z-component of the magnetization.

The current is applied perpendicular to the NIS-dipoles. After NISs have been rotated along the current, the current is switched off ($j = 0$ for $t > 35$ns). This leads to the repulsive interaction between NISs [27].

The considered effect is based on the anisotropic NIS-NIS interaction potential [27]: NISs attract each other being oriented head-to-head (initial configuration in the Supplementary Movie) and repulse being oriented side-to-side (the configuration after $t = 35$ns). Thus, we stress that the SPC may disassemble the coupled pair of NISs or vice versa couples remote skyrmions into skyrmion chains. We also show that NISs rotate the surrounding homogeneous state, which otherwise is insensitive to SPC. Thus, NISs could be utilized as tumblers in nanostructures that rotate the surrounding obliqie phases.
